# The Association Between Triglyceride-Glucose Index and Mortality Risk in Cardiovascular Disease Patients: A Meta-Analysis

**DOI:** 10.14740/jocmr6567

**Published:** 2026-06-30

**Authors:** Ren Jie Wu, Jie Yu, Ying Zhao, Jie Han, Xing Liang Shi, Jia Bing Wang, Ming Wu Hu, Ting Li

**Affiliations:** a Department of Cardiovascular Diseases, Hangzhou Xiaoshan Second People’s Hospital, Xiaoshan District, Hangzhou, Zhejiang Province, China; b Hangzhou Ninth People’s Hospital, Hangzhou, Zhejiang Province, China

**Keywords:** Meta-analysis, Mortality, Cardiovascular disease, Triglyceride-glucose index, Prognostic marker, Type 2 diabetes mellitus

## Abstract

**Background:**

The triglyceride-glucose (TyG) index was proposed as a possible indicator with regard to projecting cardiovascular disease (CVD) outcomes. Nevertheless, its capacity to project outcomes in various CVD patient groups, particularly those without type 2 diabetes mellitus (T2DM), remains uncertain. This meta-analysis is intended to assess the relationship that exists between baseline TyG levels and mortality outcomes in hospitalized CVD patients while examining potential variations in this association across different subgroups.

**Methods:**

The authors systematically searched the Embase, PubMed and Web of Science databases for studies evaluating the connection between TyG levels and mortality outcomes in CVD patients. Seventeen studies, including a total of 26,464 participants, were included. The primary findings analyzed were all-cause mortality and cardiovascular mortality. Stratified and sensitivity analyses were performed to evaluate the robustness and generalizability of the findings.

**Results:**

Elevated TyG levels were significantly associated with higher risks of both all-cause mortality (hazard ratio (HR) = 1.43; 95% CI, 1.27–1.62; P < 0.001) and cardiovascular mortality (HR = 1.62; 95% CI, 1.33–1.97; P < 0.001) in patients with acute and chronic CVD. However, this association was not significant in patients without T2DM. Dose-response meta-analysis indicated a linear increase in all-cause mortality risk with higher TyG levels. Sensitivity analyses confirmed the robustness of the results, and no significant publication bias was detected.

**Conclusions:**

TyG could potentially predict mortality risk in CVD patients, particularly within the Asian population. Additional high-quality, large-scale studies across diverse ethnic groups (such as Caucasians) are needed to validate these results’ global generalizability.

## Introduction

Cardiovascular diseases (CVD) represent the primary driver of global morbidity and mortality, imposing substantial clinical and socioeconomic burdens on healthcare systems [1]. Although advancements in percutaneous coronary intervention (PCI) and novel pharmacotherapies have significantly improved acute outcomes, hospitalized CVD patients still face a high residual risk of recurrent ischemic events and long-term mortality [2]. Therefore, identifying accessible, cost-effective biomarkers for precise risk stratification remains a clinical priority.

Insulin resistance (IR) is well-established as a core pathophysiological driver in the initiation, progression, and destabilization of atherosclerotic plaques [3]. Mechanistically, IR accelerates atherogenesis through a cascade of interrelated pathways, including endothelial dysfunction, heightened oxidative stress, persistent systemic inflammation, and the induction of a prothrombotic state. Consequently, integrating IR assessment into clinical practice could substantially improve prognosis prediction in CVD patients.

The hyperinsulinemic-euglycemic clamp remains the gold standard for quantifying IR, while the homeostasis model assessment of insulin resistance (HOMA-IR) is widely utilized in epidemiological studies [4]. However, both methods rely on fasting insulin quantification, which faces limitations in acute or emergency cardiovascular settings due to high costs, lack of routine laboratory availability, and inter-assay variability [5].

To address these limitations, the triglyceride-glucose (TyG) index—derived from fasting triglycerides (TG) and fasting blood glucose (FBG)—has emerged as a reliable, inexpensive surrogate marker for IR [6]. Notably, the prognostic value of the TyG index extends beyond that of its individual components. While TG and FBG individually reflect isolated metabolic disturbances, the TyG index captures the synergistic pathological impact of concurrent “lipotoxicity” and “glucotoxicity” [7]. By reflecting the combined burden of ectopic lipid deposition and impaired glucose homeostasis at the tissue level, the TyG index serves as a more comprehensive predictive tool for cardiovascular outcomes than TG or FBG alone.

Previous research has explored the relationship between the TyG index and the prognosis of patients with CVD [8–11]. However, the results have been inconsistent. On the other hand, a meta-analysis, for instance, one study categorized the TyG index into high as well as low groups and found no significant link between cardiovascular and TyG levels or all-cause mortality in patients with coronary artery disease (CAD) [12]. Nevertheless, other studies have suggested that this relationship may be non-linear [13, 14]. Hence, it is crucial to evaluate the most recent research by integrating conventional meta-analysis with dose-response meta-analysis to evaluate the relationship that exists between baseline TyG levels and mortality risk in CVD patients.

## Materials and Methods

### Search strategy

This study conducted a literature search following a predetermined strategy employing databases like Web of Science, Embase, and PubMed. Moreover, the search keywords included “cardiovascular disease,” “coronary heart disease,” “acute coronary syndrome,” “acute heart failure,” “chronic heart failure,” “triglyceride-glucose index,” “TyG,” and “mortality.” The search was current as of May 6, 2025, and no language restrictions were applied. Other than that, prevalent references as well as reviews from the included studies were examined to assess additional research suitable with regard to meta-analysis.

### Literature selection

The criteria for including studies in this analysis were given as follows: (1) The subjects were CVD patients receiving hospital treatment (for example, coronary heart disease, heart failure, myocardial infarction, acute coronary syndrome), including both acute and chronic cases; (2) The studies reported baseline TyG levels in CVD patients, with subjects divided into at least two categories referring to their TyG levels; (3) The studies reported the connection between TyG and follow-up CVD mortality or all-cause mortality, assessing whether TyG was substantially related to prognostic outcomes using hazard ratio (HR) and 95% confidence interval (CI); and (4) This paper design was either a retrospective or prospective or cohort study.

The exclusion criteria were: (1) Non-original research, including conference abstracts, reviews, as well as commentaries; (2) Studies reporting in-hospital mortality as the outcome; (3) Duplicate publications or multiple articles based on the same dataset, in which case only the most comprehensive study was included, while the others were omitted.

### Data extraction and quality assessment

Two investigators independently reviewed the literature based on the exclusion and inclusion criteria. Upon completion of the studies for analysis, data extraction was independently conducted by the two investigators employing a pre-structured template. Furthermore, the retrieved data encompassed study region, publication year, first author, study design, subjects essential characteristics (age, sample size, gender composition, body mass index (BMI)), follow-up period, and study outcomes. After completing data collection, the two investigators shared and reviewed the extraction forms, and any discrepancies were clarified through discussion.

The methodological quality of the included studies was examined employing the Newcastle-Ottawa Scale (NOS), which covers three factors: exposure (with a total of eight scoring items and a maximum score of 9) [15], comparability, and study subjects’ selection. Research scoring 7–9 points were classified as high-quality, 4–6 points as medium-quality, while fewer than 4 points as low-quality.

### Statistical analysis

First, we employed HR (95% CI) representing the impact size to assess the association between TyG levels (high vs. low) and all-cause mortality as well as CVD mortality. Furthermore, heterogeneity was evaluated employing Cochran’s Q test as well as the I^2^ statistic1. If the Q statistic showed P < 0.05 or I^2^ > 50%, it highlighted substantial statistical heterogeneity between studies. If P ≥ 0.05 and I^2^ ≤ 50%, the heterogeneity was insignificant. Due to significant heterogeneity in study designs, inclusion criteria, and other aspects among the included studies, a random-effects model was implemented to combine effect sizes. Consequently, subgroup analyses were conducted to evaluate whether the connection between TyG levels, all-cause mortality, as well as CVD mortality, was significant in specific populations, such as acute CVD, chronic CVD, CVD with T2DM, and CVD without T2DM.

For studies that categorized TyG levels into at least three groups, a dose-response meta-analysis was implemented if they adhered to the listed standards: 1) HR (95% CI) was reported for each dose group; 2) Reported person-years and death counts for each group, or these can be derived from other data. The dose-response meta-analysis employed the least squares trend estimation technique constructed by Greenland et al [16] and Orsini et al [17].

Furthermore, Egger’s test was implemented to evaluate whether there was significant publication bias among the studies [18]. Meanwhile, sensitivity analysis was performed using the leave-one-out technique to examine each study’s effect on the meta-analysis results. All statistical analyses were conducted utilizing Stata 14.0 software.

### Registration

This study has been registered in PROSPERO with the registration number CRD42024505518.

### Institutional Review Board (IRB) approval

In accordance with standard international guidelines for secondary data analyses and meta-analyses, Institutional Review Board (IRB) approval was not required because this study did not involve the direct collection or modification of individual patient-level data.

### Ethical compliance with human study

Not applicable. This study is a meta-analysis of previously published data and did not involve any original research with human participants. Ethical approval and informed consent were obtained by the investigators of the primary studies, as appropriate, in accordance with the Declaration of Helsinki.

## Results

### Literature search

**Figure 1:**
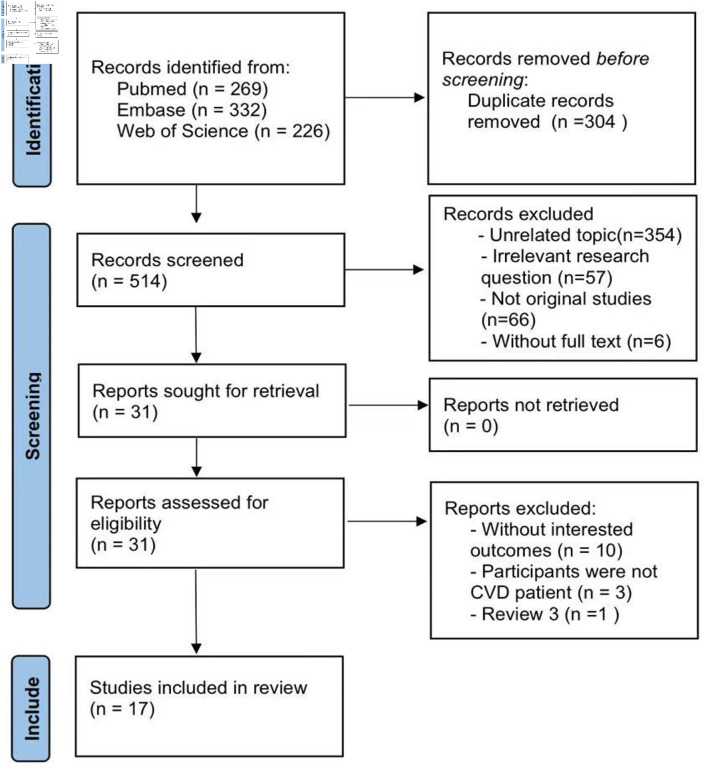
Flowchart. CVD: cardiovascular disease.

Figure 1 illustrates the results with regard to the literature review and the screening process. Initially, 818 articles were identified from electronic databases. Correspondingly, after removing 304 duplicates, 514 articles were left. After screening the abstracts and titles, 483 articles were excluded because they did not meet the inclusion criteria. Finally, after reading the full text of 31 articles, 14 were omitted, leaving 17 articles for inclusion in the meta-analysis [8–11, 13, 14, 19–29].

### Study characteristics and quality assessment

This meta-analysis included 17 studies conducted in China and Turkey. Three studies were prospective [13, 21, 23], while the remainder were retrospective cohort studies. The study sample sizes varied from 231 to 6,697, yielding a total of 26,464 participants (18,393 men and 8,071 women). Detailed information on participants’ age, BMI, type of CVD, and whether they had concurrent diabetes are provided in Table 1 [8–11, 13, 14, 19–29].

**Table 1:** Characteristics With Regard to the Seventeen Included Studies

Study	Country	Design	Patients	Acute, %	Cut-off for TyG	N, M/F	Age, years	BMI, kg/m^2^	T2DM, %	Follow-up, years
Guo et al, 2021 [19]	China	RCS	CHF	0	Tertiles	546, 362/184	65.18 (12.01)	21.07 (1.83)	100	1, median
Hao et al, 2024 [13]	China	PCS	CHD	74.6	Quintile	3,321, 2,404/917	61.7 ± 11.7	25.6 ± 3.5	32.4	9.79, median
Hao et al, 2023 [14]	China	RCS	AMI	100	Tertiles	1,144, 902/242	62.1 ± 12.8	24.8 ± 3.3	18.9	1
Huang et al, 2022 [20]	China	RCS	AHF	100	Tertiles	932, 579/353	70 (61, 80)^a^	24.2 (21.6, 27.1)^a^	32.8	1.31
Jiao et al, 2022 [21]	China	PCS	ACS	100	Tertiles	662, 476/186	81.87 ± 2.14	24.57 ± 3.40	34.9	≤ 10
Mao et al, 2019 [8]	China	RCS	NSTE-ACS	100	Median	438, 295/143	62.5 (53.0, 68.0)^a^	24 33 ± 3 17	32.6	1
Ozcan et al, 2023 [22]	Turkey	RCS	HF	NR	Tertiles	773, 633/140	63 (53, 72)^a^	NR	35.6	3.17
Shen et al, 2023 [23]	China	PCS	ACS	100	Tertiles	231, 156/75	81.58 ± 1.93	24.78 ± 3.42	100	4.08
Sun et al, 2023 [24]	China	RCS	iHF	0	Quartiles	2,055, 1,690/365	60.3 ± 11.0	25.9 ± 3.2	38.5	3
Wang et al, 2020 [25]	China	RCS	ACS	100	Tertiles	2,531, 1,415/1,116	66.3 ± 6.8	25.9 ± 2.7	100	3
Xie et al, 2023 [26]	China	RCS	CHD	59.2	Tertiles	1,061, 789/272	61.8 ± 10.5	NR	52.7	1.83
Zhang et al, 2021 [9]	China	RCS	AMI	100	Tertiles	1,932, 1,324/608	65.4 ± 12.0	25.8 ± 3.5	100	2.23
Zhang et al, 2022 [27]	China	RCS	ACS	100	Median	1,010, 735/275	65.8 ± 10.1	25.6 ± 3.4	0	2.97
Zhao et al, 2020 [28]	China	RCS	NSTE-ACS	100	ROC	798, 545/253	60.9 ± 8.3	26.7 ± 3.2	100	3
Zhao, et al, 2021 [10]	China	RCS	NSTE-ACS	100	Median	1,510, 1,113/397	59.7 ± 9.3	25.8 ± 3.1	0	4
Zhou et al, 2023 [11]	China	RCS	AHF	100	Tertiles	823, 396/427	73.0 ± 12.7	25.5 ± 4.7	42	3.16
Zhou et al, 2023 [29]	China	RCS	CHF	0	Tertiles	6,697, 4,579/2,118	63.3 ± 14.2	25.2 (22.8, 27.8)^a^	44.6	3.9

^a^Data are expressed as median (interquartile range). ACS: acute coronary syndrome; AHF: acute heart failure; AMI: acute myocardial infarction; BMI: body mass index; CHD: coronary heart disease; CHF: chronic heart failure; HF: heart failure; iHF: ischemic heart failure; M/F: male/female; NR: not reported; NSTE-ACS: non-ST-segment elevation acute coronary syndrome; PCS: prospective cohort study; RCS: retrospective cohort study; ROC: receiver operating characteristic; T2DM: type 2 diabetes mellitus; TyG: triglyceride-glucose index.

The quality assessment results (Table 2) [8–11, 13, 14, 19–29] indicated some degree of bias in participant selection and follow-up duration. Overall, the methodological quality of one study was rated as “moderate”. Meanwhile, the others were rated as “high”.

**Table 2:** Quality Assessment of the Cohort Studies With Newcastle-Ottawa Quality Assessment Scale

Study	Representativeness of the exposed cohort	Selection of the unexposed cohort	Ascertainment of exposure	Outcome of interest not present at start of study	Control for important factor or additional factor	Outcome assessment	Follow-up long enough for outcomes to occur	Adequacy of follow-up of cohorts	Total quality scores
Guo et al, 2021 [19]	☆	☆	☆	—	—	☆	—	☆	5
Hao et al, 2024 [13]	☆	☆	☆	—	☆☆	☆	☆	☆	8
Hao et al, 2023 [14]	☆	☆	☆	☆	☆☆	☆	—	☆	8
Huang et al, 2022 [20]	☆	☆	☆	—	☆☆	☆	—	☆	7
Jiao et al, 2022 [21]	☆	☆	☆	☆	☆☆	☆	—	☆	8
Mao et al, 2019 [8]	☆	☆	☆	—	☆☆	☆	—	☆	7
Ozcan et al, 2023 [22]	☆	☆	☆	—	☆☆	☆	—	☆	7
Shen et al, 2023 [23]	☆	☆	☆	☆	☆☆	☆	—	☆	8
Sun et al, 2023 [24]	☆	☆	☆	—	☆☆	☆	—	☆	7
Wang et al, 2020 [25]	☆	☆	☆	—	☆☆	☆	—	☆	7
Xie et al, 2023 [26]	☆	☆	☆	—	☆☆	☆	—	☆	7
Zhang et al, 2021 [9]	☆	☆	☆	—	☆☆	☆	—	☆	7
Zhang et al, 2022 [27]	☆	☆	☆	—	☆☆	☆	—	☆	7
Zhao et al, 2020 [28]	☆	☆	☆	—	☆☆	☆	—	☆	7
Zhao et al, 2021 [10]	☆	☆	☆	—	☆☆	☆	—	☆	7
Zhou et al, 2023 [11]	☆	☆	☆	—	☆☆	☆	—	☆	7
Zhou et al, 2023 [29]	☆	☆	☆	—	☆☆	☆	—	☆	7

Quality assessment was performed using the Newcastle-Ottawa Quality Assessment Scale (NOS) for cohort studies. One star (☆) indicates that one star was awarded for meeting the quality criteria for the specific item. Two stars (☆☆) indicate two stars were awarded (typically for adequate control of both the primary and additional confounding factors). Em dash (—) indicates that the study did not meet the criterion for that item and received no star.

### Meta-analysis

#### High vs. low

**Figure 2:**
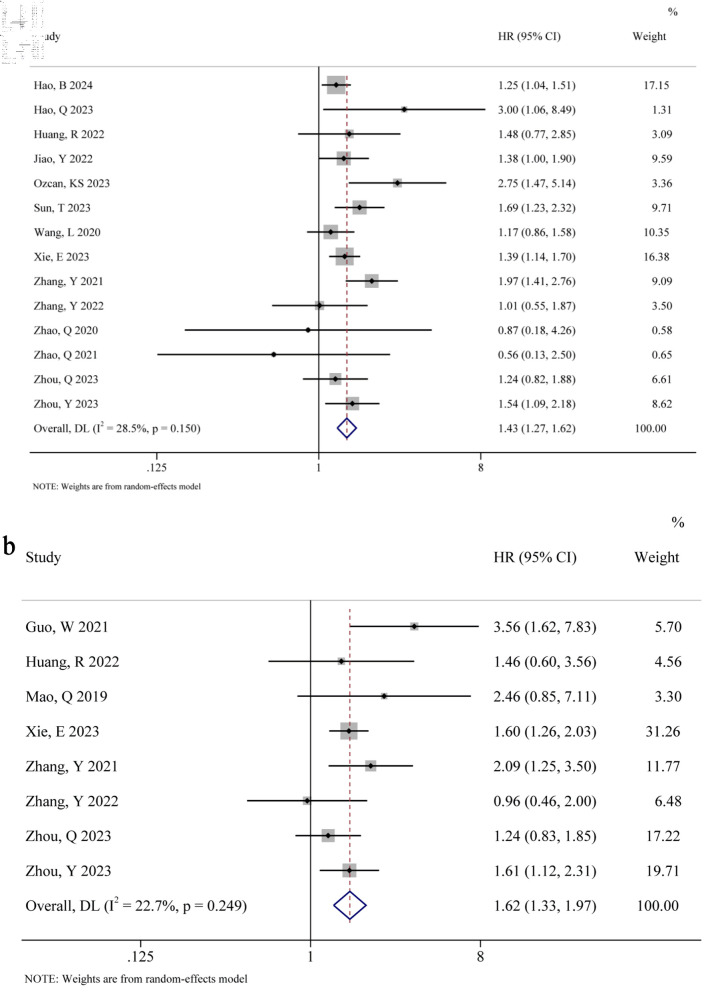
(a) All-cause mortality (high vs. low). (b) CVD mortality (high vs. low). HR: hazard ratio; CI: confidence interval.

Figure 2a, b displays the forest plots for the meta-analysis of all-cause and CVD mortality. The combined results from the random-effects model were as follows: the HR for all-cause mortality was 1.43 (95% CI, 1.27–1.62; P < 0.001), whereas the the HR for CVD mortality was 1.62 (95% CI, 1.33–1.97, P < 0.001). These results underscore the significant impact of TyG levels on mortality, particularly concerning cardiovascular outcomes. There was no substantial statistical heterogeneity among the included studies (I^2^ < 50.0%, P > 0.05), indicating good homogeneity of effect sizes.

#### Subgroup analysis

Table 3 illustrates the results of a subgroup analysis that encompasses five factors for meta-regression: country (P > 0.05), presence of acute myocardial infarction (P > 0.05), TyG cut-off point (P > 0.05), inclusion of diabetes (P > 0.05), and follow-up duration (P > 0.05). These findings suggest that these factors do not significantly contribute to heterogeneity.

**Table 3:** Subgroup Analysis

Effect size	Coefficient	Standard error	*t*	P>|t|^a^	95% confidence interval
Country	0.6861984	0.381515	1.80	0.110	−0.1935768 to 1.565974
With ACS	0.0349545	0.0891832	0.39	0.705	−0.1707022 to 0.2406113
Cut-off for TyG	–0.0165432	0.1436475	−0.12	0.911	−0.3477949 to 0.3147084
With diabetes	–0.4669322	0.3607914	−1.29	0.232	−1.298919 to 0.3650543
Follow-up	–0.12924	0.1357905	−0.95	0.369	−0.4423735 to 0.1838936
Intercept	0.2928392	0.5261956	0.56	0.593	−0.9205701 to 1.506248

^a^The two-tailed P value evaluating the statistical significance of each covariate. TyG: triglyceride-glucose; ACS: Acute coronary syndrome.

#### Dose-response meta-analysis

**Figure 3:**
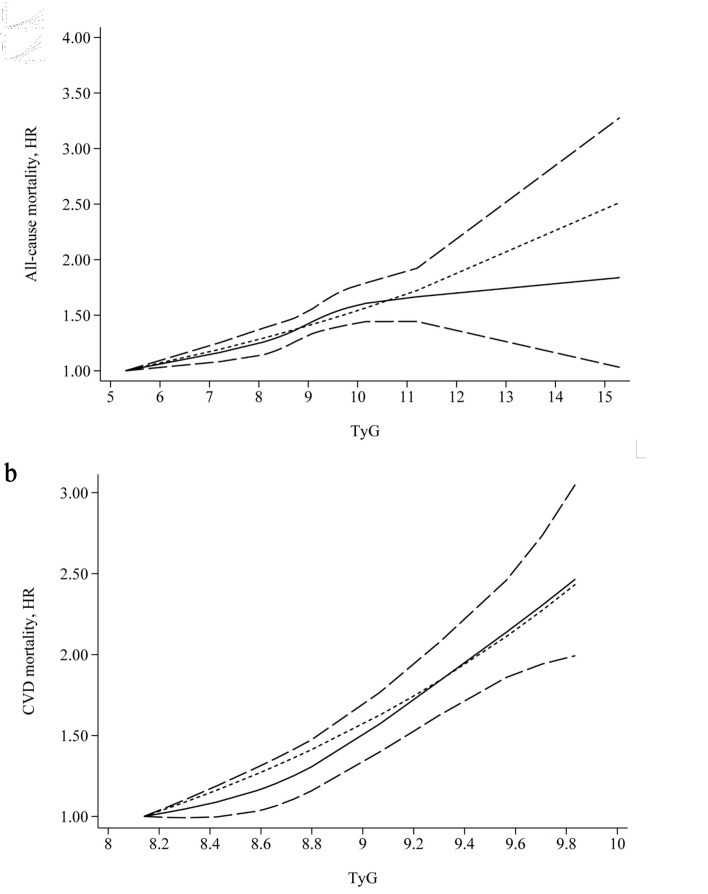
(a) All-cause-dose-response. (b) CVD- dose-response. CVD: cardiovascular disease; TyG: triglyceride-glucose.

Nine studies [9, 11, 13, 20, 21, 23–26, 29] were included in the dose-response meta-analysis evaluating the association between TyG and all-cause mortality. The linearity test yielded a P value of 0.492, suggesting no significant non-linear association between the two. The model fitting results, illustrated in Figure 3a, demonstrate an increasing trend in all-cause mortality risk with higher TyG levels. However, when TyG > 10, the width of the 95% CI began to increase, likely due to the small sample size with TyG levels greater than 10, leading to lower precision in results.

**Figure 4:**
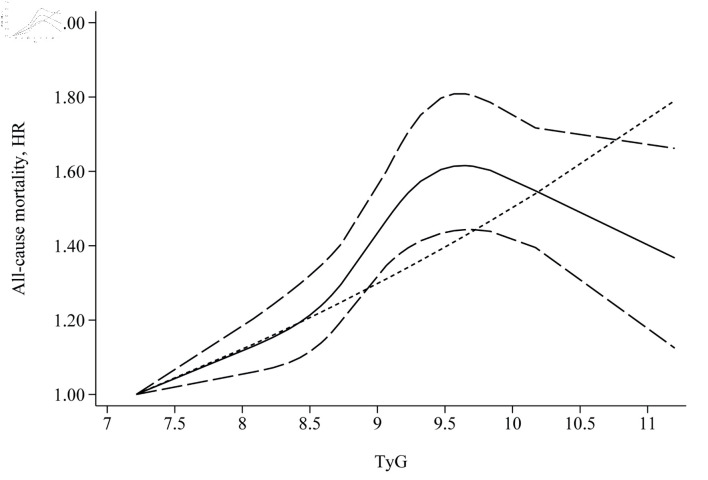
All cause-China. TyG: triglyceride-glucose.

When only studies conducted in China were included, the dose-response connection between TyG and as all-cause mortality changed (Fig. 4). The linearity test yielded a P value of 0.001, indicating a significant non-linear association. With increasing TyG levels, the effect size first increased and then decreased. However, compared to the lowest dose, any increase in TyG was correlated with a marked increase in all-cause mortality risk (P < 0.05).

Six studies [9, 11, 19, 20, 26, 29] conducted in China were included in the dose-response meta-analysis evaluating the association between TyG and CVD mortality. The linearity test yielded a P value of 0.306, indicating no substantial non-linear association. The model fitting results, shown in Figure 3b, indicated a clear upward trend in CVD mortality risk with higher TyG levels.

### Stratified analysis results

The results of the stratified analysis for all-cause mortality are presented here (Supplementary Materials 1, 2, jocmr.elmerjournals.com). Elevated TyG levels were strongly linked to a greater risk of mortality in both acute CVD and chronic CVD. However, in patients without concurrent diabetes, there was no notable correlation between TyG levels and all-cause mortality (HR = 1.76; 95% CI, 0.53–5.86; P = 0.358).

For CVD mortality, elevated TyG levels were substantially related to a greater mortality risk in acute and chronic CVD cases (Table 4; Supplementary Material 3, jocmr.elmerjournals.com). Due to the lack of HR (95% CI) data for CVD patients in both those having and not having type 2 diabetes mellitus (T2DM) in most studies, stratified analysis could not be performed.

**Table 4:** Outcomes of the Stratified Analysis

Outcomes	Number of studies	HR (95%CI)	P_A_	Heterogeneity test	
				P	I^2^ (%)
All-cause mortality	14	1.43 (1.27, 1.62)	< 0.001	0.150	28.5
Type of CVD					
Acute	9	1.38 (1.13, 1.68)	0.001	0.217	25.5
Chronic	2	1.62 (1.28, 2.05)	< 0.001	0.699	0.0
With T2DM					
Yes	5	1.50 (1.10, 2.05)	0.011	0.120	45.4
No	4	1.76 (0.53, 5.86)	0.358	< 0.001	90.9
CVD mortality	7	1.62 (1.33, 1.97)	< 0.001	0.249	22.7
Type of CVD					
Acute	5	1.47 (1.09, 1.99)	0.012	0.323	14.3
Chronic	2	2.21 (1.03, 4.73)	0.042	0.073	68.9

P_A_: P for association; it represents the statistical significance P value for the pooled effect size (HR) to test whether the HR is significantly different from 1. CVD: cardiovascular disease; T2DM: type 2 diabetes mellitus; HR: hazard ratio; CI: confidence interval.

### Publication bias test and sensitivity analysis

The Egger test’s findings indicated that both outcome measures had P values more than 0.05, implying an insignificant publication within the included studies. The leave-one-out sensitivity analysis revealed that the pooled results of all outcome measures remained materially unchanged when any single study was omitted. This indicates that the effect of individual studies on the combined findings is minimal, demonstrating good robustness of the meta-analysis results (Table 5; Supplementary Materials 4, 5, jocmr.elmerjournals.com).

**Table 5:** Outcomes With Regard to the Sensitivity Analysis and the Test of Publication Bias

Outcomes	Number of studies	Sensitivity analysis		Egger’ s test
		HR (95% CI)	Robust	P value
All-cause mortality	14	1.38 (1.23, 1.54) to 1.47 (1.29, 1.69)	Yes	0.619
CVD mortality	8	1.55 (1.32, 1.82) to 1.70 (1.38, 2.10)	Yes	0.549

CVD: cardiovascular disease; HR: hazard ratio; CI: confidence interval.

## Discussion

### Analysis and discussion of results

This meta-analysis evaluated the prognostic value of the baseline TyG index regarding adverse clinical outcomes in hospitalized patients with CVD. Pooling data from 17 high-quality clinical studies comprising 26,464 participants, our results demonstrated that an elevated baseline TyG index was significantly associated with an increased risk of adverse clinical outcomes. Notably, subgroup analysis revealed that this prognostic association was highly pronounced in patients with concurrent T2DM. These findings indicate that the baseline TyG index may serve as a potential prognostic marker for identifying high-risk individuals within the hospitalized CVD population.

A critical consideration regarding the clinical utility of the TyG index is whether it provides superior prognostic value over individual assessments of fasting TG or FBG. Our findings indicate that the TyG index possesses predictive capacity for adverse clinical outcomes, a superiority grounded in the integrated pathophysiology of its individual components. While isolated elevations in TG and FBG represent well-established independent cardiovascular risk factors, they merely reflect discrete metabolic derangements. Conversely, the TyG index functions as a composite surrogate for IR, effectively capturing the systemic nature of interconnected metabolic disorders [30]. By encapsulating this dual-pathway insult, the TyG index offers a more comprehensive evaluation of the cumulative metabolic and atherosclerotic burden than TG or FBG measured in isolation [31].

Importantly, the TyG index should be conceptualized as a distinct risk-stratification tool that supplements, rather than replaces, traditional gold-standard cardiovascular biomarkers like low-density lipoprotein cholesterol (LDL-C) and glycated hemoglobin (HbA1c). While LDL-C acts as a major driver of atherogenic plaque volume and macrovascular lipid deposition, it lacks the biological capacity to capture the metabolic inflammation that underpins residual cardiovascular risk. The TyG index, as a validated surrogate for systemic IR, reflects the underlying endothelial dysfunction, oxidative stress, and prothrombotic state that directly precipitate plaque destabilization and subsequent ischemic events. Similarly, whereas HbA1c primarily tracks cumulative glycemic load, the TyG index mathematically integrates fasting TG and glucose, thereby accounting for the synergistic tissue-level impact of concurrent lipotoxicity and glucotoxicity. This distinct pathophysiological basis explains its robust prognostic value observed even in non-diabetic populations.

For CVD patients having T2DM, the elevated TyG index reflects underlying metabolic dysfunction [32], which exacerbates the progression of atherosclerosis as well as elevates the risk of adverse cardiovascular outcomes [33]. This is likely due to the combined impacts of chronic inflammation, dyslipidemia, and IR, all of which are more pronounced in patients having T2DM [34–36].

The differential impact of TyG levels on CVD prognosis in patients with and without T2DM may be attributed to the underlying pathophysiological differences between these groups. In T2DM patients, IR is a central feature that directly contributes to endothelial dysfunction, hypercoagulability, and accelerated atherosclerosis [36, 37]. On the other hand, the TyG index, acting as a proxy indicator with regard to IR, thus serves as a more direct indicator of cardiovascular risk in these individuals [38, 39]. Conversely, in non-diabetic patients, the relationship between cardiovascular as well as IR (as indicated by TyG) outcomes might be modulated by other factors (such as advanced age, chronic hypertension, smoking habits, or genetic predispositions to vascular calcification), reducing the overall predictive power of the TyG index [40].

Moreover, the differential prognostic performance of the TyG index across various patient populations may be deeply intertwined with early subclinical myocardial changes. In patients with metabolic disorders such as T2DM, IR induces lipotoxicity, chronic inflammation, and microvascular impairment, culminating in subtle myocardial dysfunction. In this clinical context, myocardial strain imaging—particularly global longitudinal strain (GLS)—has emerged as a powerful and established tool for the early detection of subclinical myocardial impairment, demonstrating high sensitivity even in patients with preserved ejection fraction [41]. Given that myocardial strain parameters are independently associated with long-term adverse cardiovascular outcomes and mortality [42], integrating metabolic indicators with advanced imaging modalities could significantly refine current risk-stratification models. Future high-quality studies are strongly warranted to explore the direct correlation between the TyG index and myocardial strain parameters. Such investigations will help clarify whether a high TyG index can serve as an accessible metabolic surrogate for subclinical myocardial dysfunction, thereby expanding its clinical utility in personalized cardiovascular care.

These results have important clinical implications. Concerning CVD patients with T2DM, regular monitoring of TyG levels could be crucial in stratifying risk and guiding more aggressive management strategies [43, 44]. Interventions to reduce IR, such as lifestyle modification and pharmacotherapy, might be particularly beneficial in this group. On the other hand, for CVD patients without T2DM, while TyG may still provide some prognostic information, it should be considered alongside a broader range of risk factors when making clinical decisions.

### Limitations of the study

There was significant variability in methodology and clinical characteristics among the included studies, although statistical heterogeneity was low. Nonetheless, the consistency with regard to the meta-analysis results implies that the link between TyG levels and mortality risk may apply to all types of CVD patients.

Due to the reliance on study-level aggregated data, we could not standardize the exact fasting durations for triglyceride measurements across all cohorts, nor could we control for crucial lifestyle confounders, such as diet (particularly sugar and carbohydrate intake), physical activity, and alcohol consumption, which are well-known to modulate both TyG levels and mortality risk. Although our use of a random-effects model helps mitigate statistical heterogeneity, residual confounding from these unmeasured factors cannot be entirely ruled out.

Some studies adjusted for the impact of treatment regimens on outcomes. However, the influence of intervention strategies on the results cannot be ignored. Other than that, there was insufficient information to perform a stratified analysis based on intervention strategies.

The studies included were mainly conducted in China and Turkey, emphasizing the need for more research from other regions to analyze the relationship between TyG levels and CVD.

### Conclusions

TyG may serve as a benchmark with regard to mortality risk in CVD patients, particularly for Asian populations. Given the limited representation of Caucasian participants in current literature, further large-scale, high-quality research in Western and multi-ethnic cohorts is recommended to verify the global generalizability of these results.

## Supplementary Material

Suppl 1 All-cause subgroup.

Suppl 2 All-cause diabetes subgroup.

Suppl 3 CVD subgroup.

Suppl 4 All-cause mortality.

Suppl 5 CVD mortality.

## Data Availability

All data generated or analyzed during this study are included in this published article.

## Abbreviations

TyG: triglyceride-glucose
CVD: cardiovascular disease
CAD: coronary artery disease
T2DM: type 2 diabetes mellitus
HR: hazard ratio
NOS: Newcastle-Ottawa Scale

## References

[1] Shaw LJ, Blankstein R, Bax JJ, Ferencik M, Bittencourt MS, Min JK, Berman DS (2021). Society of cardiovascular computed tomography / North American society of cardiovascular imaging - expert consensus document on coronary CT imaging of atherosclerotic plaque. J Cardiovasc Comput Tomogr.

[2] Bhatt DL, Steg PG, Miller M, Brinton EA, Jacobson TA, Ketchum SB, Doyle RT (2019). Cardiovascular risk reduction with icosapent ethyl for hypertriglyceridemia. N Engl J Med.

[3] Bornfeldt KE, Tabas I (2011). Insulin resistance, hyperglycemia, and atherosclerosis. Cell Metab.

[4] Matthews DR, Hosker JP, Rudenski AS, Naylor BA, Treacher DF, Turner RC (1985). Homeostasis model assessment: insulin resistance and beta-cell function from fasting plasma glucose and insulin concentrations in man. Diabetologia.

[5] Sacks DB, Arnold M, Bakris GL, Bruns DE, Horvath AR, Kirkman MS, Lernmark A (2011). Guidelines and recommendations for laboratory analysis in the diagnosis and management of diabetes mellitus. Diabetes Care.

[6] Simental-Mendia LE, Rodriguez-Moran M, Guerrero-Romero F (2008). The product of fasting glucose and triglycerides as surrogate for identifying insulin resistance in apparently healthy subjects. Metab Syndr Relat Disord.

[7] Hong S, Han K, Park CY (2020). The triglyceride glucose index is a simple and low-cost marker associated with atherosclerotic cardiovascular disease: a population-based study. BMC Med.

[8] Mao Q, Zhou D, Li Y, Wang Y, Xu SC, Zhao XH (2019). The triglyceride-glucose index predicts coronary artery disease severity and cardiovascular outcomes in patients with non-ST-segment elevation acute coronary syndrome. Dis Markers.

[9] Zhang Y, Ding X, Hua B, Liu Q, Gao H, Chen H, Zhao XQ (2021). Predictive effect of triglyceride-glucose index on clinical events in patients with type 2 diabetes mellitus and acute myocardial infarction: results from an observational cohort study in China. Cardiovasc Diabetol.

[10] Zhao Q, Zhang TY, Cheng YJ, Ma Y, Xu YK, Yang JQ, Zhou YJ (2021). Triglyceride-glucose index as a surrogate marker of insulin resistance for predicting cardiovascular outcomes in nondiabetic patients with non-st-segment elevation acute coronary syndrome undergoing percutaneous coronary intervention. J Atheroscler Thromb.

[11] Zhou Q, Yang J, Tang H, Guo Z, Dong W, Wang Y, Meng X (2023). High triglyceride-glucose (TyG) index is associated with poor prognosis of heart failure with preserved ejection fraction. Cardiovasc Diabetol.

[12] Luo JW, Duan WH, Yu YQ, Song L, Shi DZ (2021). Prognostic significance of triglyceride-glucose index for adverse cardiovascular events in patients with coronary artery disease: a systematic review and meta-analysis. Front Cardiovasc Med.

[13] Hao B, Lyu L, Xu J, Zhu X, Xu C, Gao W, Qin J (2024). The relationship between triglyceride-glucose index and prospective key clinical outcomes in patients hospitalised for coronary artery disease. Cardiovasc Diabetol.

[14] Hao Q, Yuanyuan Z, Lijuan C (2023). The prognostic value of the triglyceride glucose index in patients with acute myocardial infarction. J Cardiovasc Pharmacol Ther.

[15] Wells GA, Wells G, Shea B, Shea B, O'Connell D, Peterson J

[16] Greenland S, Longnecker MP (1992). Methods for trend estimation from summarized dose-response data, with applications to meta-analysis. Am J Epidemiol.

[17] Orsini N, Li R, Wolk A, Khudyakov P, Spiegelman D (2012). Meta-analysis for linear and nonlinear dose-response relations: examples, an evaluation of approximations, and software. Am J Epidemiol.

[18] Egger M, Davey Smith G, Schneider M, Minder C (1997). Bias in meta-analysis detected by a simple, graphical test. BMJ.

[19] Guo W, Zhao L, Mo F, Peng C, Li L, Xu Y, Guo W (2021). The prognostic value of the triglyceride glucose index in patients with chronic heart failure and type 2 diabetes: A retrospective cohort study. Diabetes Res Clin Pract.

[20] Huang R, Wang Z, Chen J, Bao X, Xu N, Guo S, Gu R (2022). Prognostic value of triglyceride glucose (TyG) index in patients with acute decompensated heart failure. Cardiovasc Diabetol.

[21] Jiao Y, Su Y, Shen J, Hou X, Li Y, Wang J, Liu B (2022). Evaluation of the long-term prognostic ability of triglyceride-glucose index for elderly acute coronary syndrome patients: a cohort study. Cardiovasc Diabetol.

[22] Ozcan KS, Hayiroglu MI, Cinar T (2023). Admission triglyceride-glucose index is predictor of long-term mortality and appropriate implantable cardiac defibrillator therapy in patients with heart failure. Biomark Med.

[23] Shen J, Feng B, Fan L, Jiao Y, Li Y, Liu H, Hou X (2023). Triglyceride glucose index predicts all-cause mortality in oldest-old patients with acute coronary syndrome and diabetes mellitus. BMC Geriatr.

[24] Sun T, Huang X, Zhang B, Ma M, Chen Z, Zhao Z, Zhou Y (2023). Prognostic significance of the triglyceride-glucose index for patients with ischemic heart failure after percutaneous coronary intervention. Front Endocrinol (Lausanne).

[25] Wang L, Cong HL, Zhang JX, Hu YC, Wei A, Zhang YY, Yang H (2020). Triglyceride-glucose index predicts adverse cardiovascular events in patients with diabetes and acute coronary syndrome. Cardiovasc Diabetol.

[26] Xie E, Ye Z, Wu Y, Zhao X, Li Y, Shen N, Guo X (2023). Association of triglyceride-glucose index with coronary severity and mortality in patients on dialysis with coronary artery disease. Eur J Med Res.

[27] Zhang Y, Ding X, Hua B, Liu Q, Gao H, Chen H, Zhao XQ (2022). High triglyceride-glucose index is associated with poor cardiovascular outcomes in nondiabetic patients with ACS with LDL-C below 1.8 mmol/L. J Atheroscler Thromb.

[28] Zhao Q, Zhang TY, Cheng YJ, Ma Y, Xu YK, Yang JQ, Zhou YJ (2020). Impacts of triglyceride-glucose index on prognosis of patients with type 2 diabetes mellitus and non-ST-segment elevation acute coronary syndrome: results from an observational cohort study in China. Cardiovasc Diabetol.

[29] Zhou Y, Wang C, Che H, Cheng L, Zhu D, Rao C, Zhong Q (2023). Association between the triglyceride-glucose index and the risk of mortality among patients with chronic heart failure: results from a retrospective cohort study in China. Cardiovasc Diabetol.

[30] Guerrero-Romero F, Simental-Mendia LE, Gonzalez-Ortiz M, Martinez-Abundis E, Ramos-Zavala MG, Hernandez-Gonzalez SO, Jacques-Camarena O (2010). The product of triglycerides and glucose, a simple measure of insulin sensitivity. Comparison with the euglycemic-hyperinsulinemic clamp. J Clin Endocrinol Metab.

[31] Moon JH, Kim Y, Oh TJ, Moon JH, Kwak SH, Park KS, Jang HC (2023). Triglyceride-glucose index predicts future atherosclerotic cardiovascular diseases: a 16-year follow-up in a prospective, community-dwelling cohort study. Endocrinol Metab (Seoul).

[32] Li M, Zhu P, Wang SX (2022). Risk for cardiovascular death associated with waist circumference and diabetes: a 9-year prospective study in the Wan Shou Lu Cohort. Front Cardiovasc Med.

[33] Zhang H, Wang L, Zhang Q, Song Y, Cai M, Bao J, Yu Q (2024). Non-linear association of triglyceride-glucose index with cardiovascular and all-cause mortality in T2DM patients with diabetic kidney disease: NHANES 2001-2018 retrospective cohort study. Lipids Health Dis.

[34] Beverly JK, Budoff MJ (2020). Atherosclerosis: Pathophysiology of insulin resistance, hyperglycemia, hyperlipidemia, and inflammation. J Diabetes.

[35] Song Y, Zhang J, Yuan H, Zhao P (2024). An overview of the application and potential mechanism on the triglyceride glucose index with multi-vessel coronary disease. Lipids Health Dis.

[36] Boccatonda A, Del Cane L, Marola L, D'Ardes D, Lessiani G, di Gregorio N, Ferri C (2024). Platelet, antiplatelet therapy and metabolic dysfunction-associated steatotic liver disease: a narrative review. Life (Basel).

[37] Yan T, Huang Y, Wu JHY, Zhuang XD, Pan XF (2022). Editorial: insulin resistance, metabolic syndrome, and cardiovascular disease. Front Cardiovasc Med.

[38] Sun Y, Xu H, Gao W, Deng J, Song X, Li J, Liu X (2024). S100a8/A9 proteins: critical regulators of inflammation in cardiovascular diseases. Front Cardiovasc Med.

[39] Wang L, Cong HL, Zhang JX, Li XM, Hu YC, Wang C, Lang JC (2022). Prognostic performance of multiple biomarkers in patients with acute coronary syndrome without standard cardiovascular risk factors. Front Cardiovasc Med.

[40] Tao LC, Xu JN, Wang TT, Hua F, Li JJ (2022). Triglyceride-glucose index as a marker in cardiovascular diseases: landscape and limitations. Cardiovasc Diabetol.

[41] Sonaglioni A, Bordoni T, Naselli A, Nicolosi GL, Grasso E, Bianchi S, Ferrulli A (2024). Influence of gestational diabetes mellitus on subclinical myocardial dysfunction during pregnancy: A systematic review and meta-analysis. Eur J Obstet Gynecol Reprod Biol.

[42] Liu JH, Chen Y, Yuen M, Zhen Z, Chan CW, Lam KS, Tse HF (2016). Incremental prognostic value of global longitudinal strain in patients with type 2 diabetes mellitus. Cardiovasc Diabetol.

[43] Toupin S, Pezel T, Sanguineti F, Kinnel M, Hovasse T, Unterseeh T, Champagne S (2022). Additional prognostic value of stress cardiovascular magnetic resonance for cardiovascular risk stratification after a cryptogenic ischemic stroke. Front Cardiovasc Med.

[44] Zhang S, Ma Q, Jiao Y, Wu J, Yu T, Hou Y, Sun Z (2022). Prognostic value of myocardial salvage index assessed by cardiovascular magnetic resonance in reperfused ST-segment elevation myocardial infarction. Front Cardiovasc Med.

