# Supplementary figures and images for: The Association Between Triglyceride-Glucose Index and Mortality Risk in Cardiovascular Disease Patients: A Meta-Analysis
Source: J Clin Med Res. 2026 Jun 30;18(6):407–19. doi: 10.14740/jocmr6567 (PMC13375415; doi:10.14740/jocmr6567)

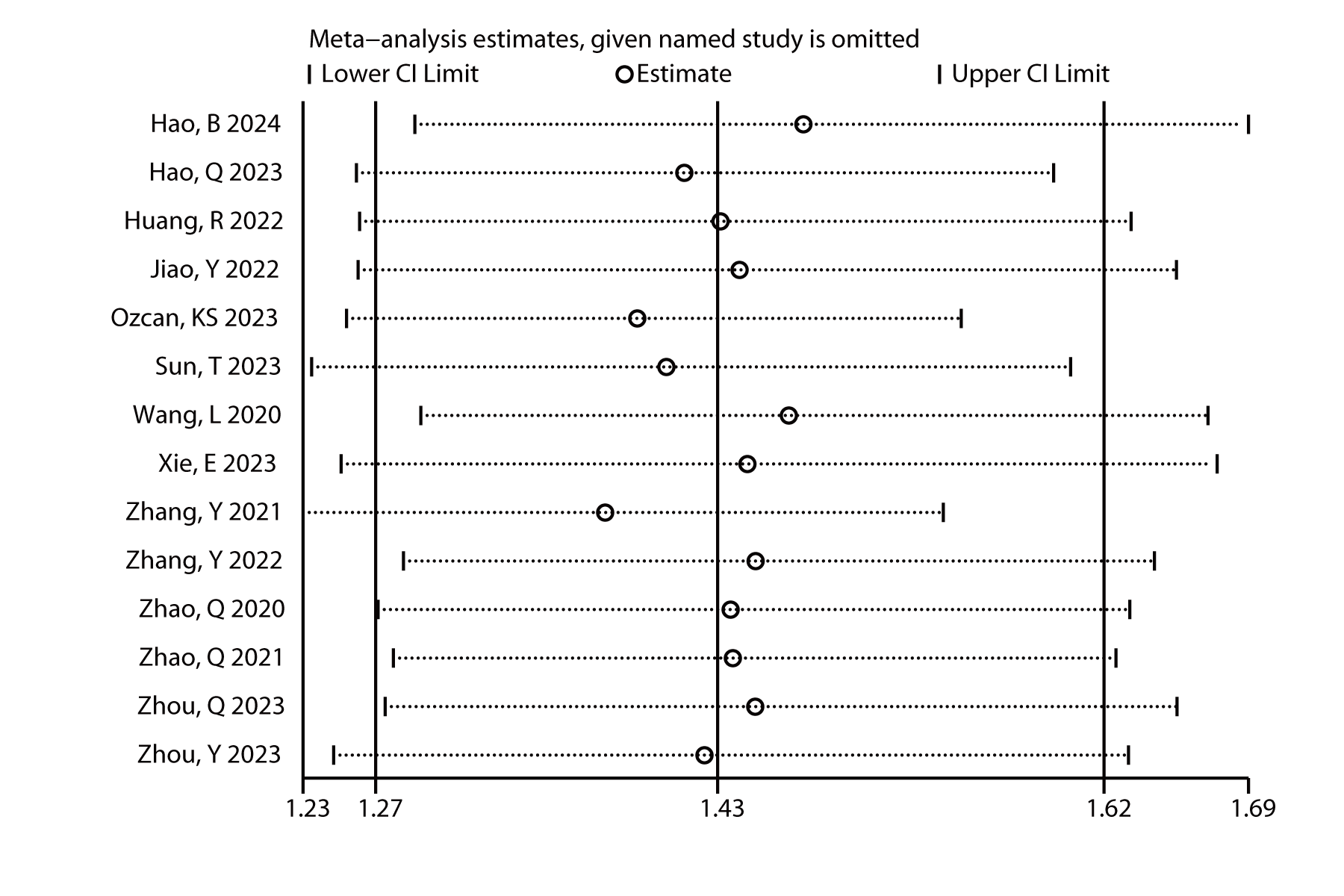


**Suppl 1.** All-cause mortality

Supplement: Suppl 1 — All-cause subgroup. [file jocmr-18-06-407-s001.docx]

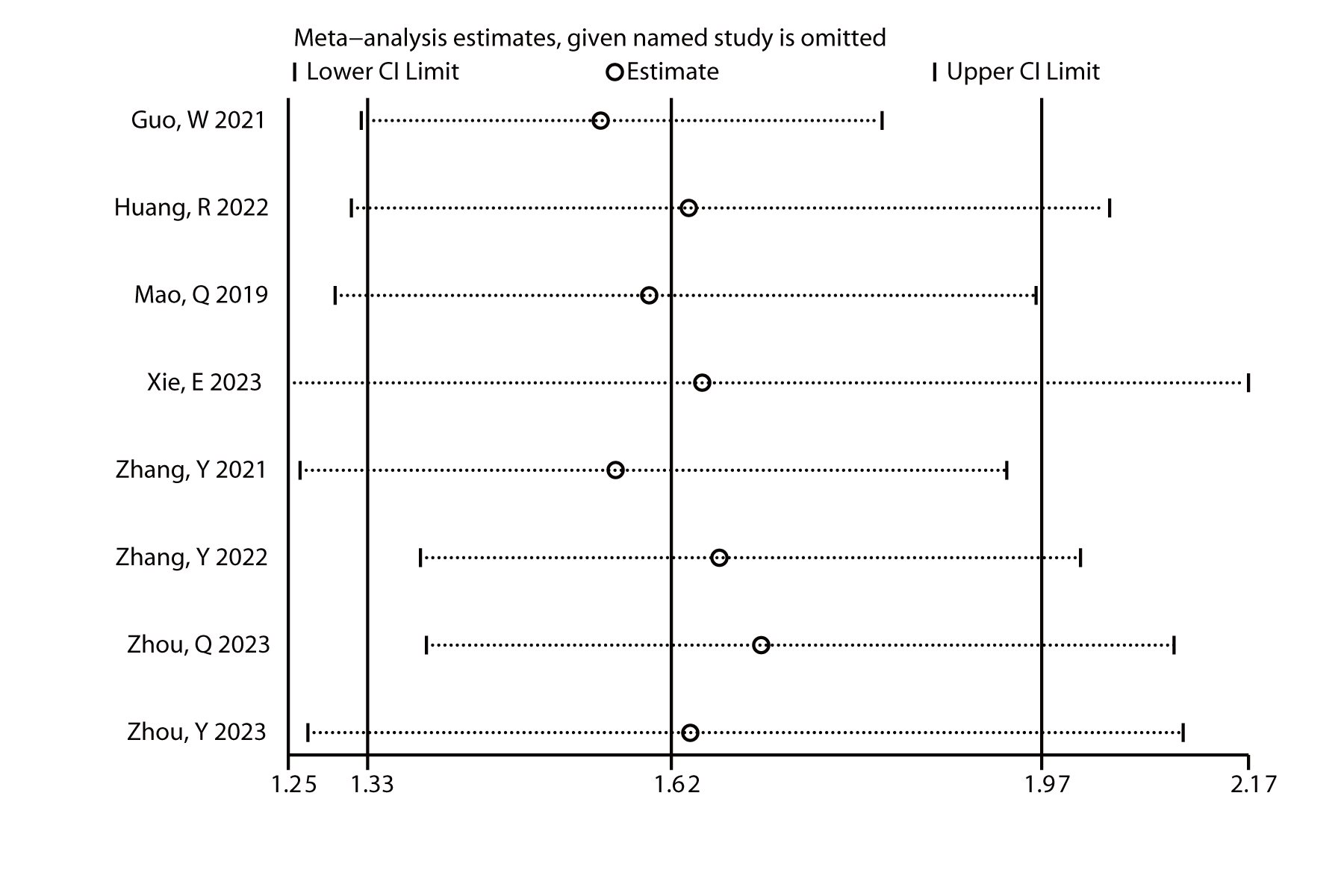


**Suppl 2.** CVD mortality

Supplement: Suppl 2 — All-cause diabetes subgroup. [file jocmr-18-06-407-s002.docx]

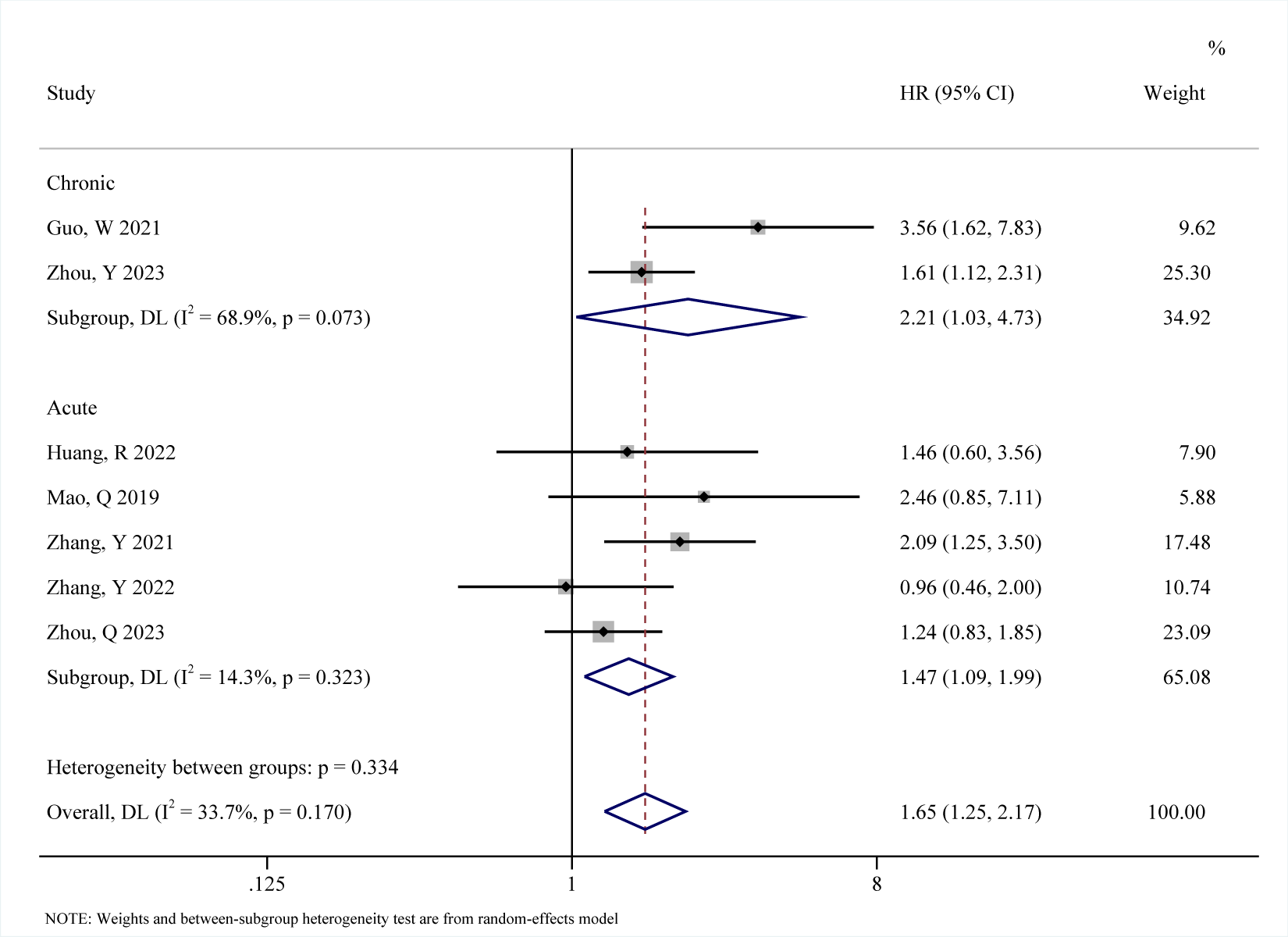


**Suppl 3.** CVD – subgroup

Supplement: Suppl 3 — CVD subgroup. [file jocmr-18-06-407-s003.docx]

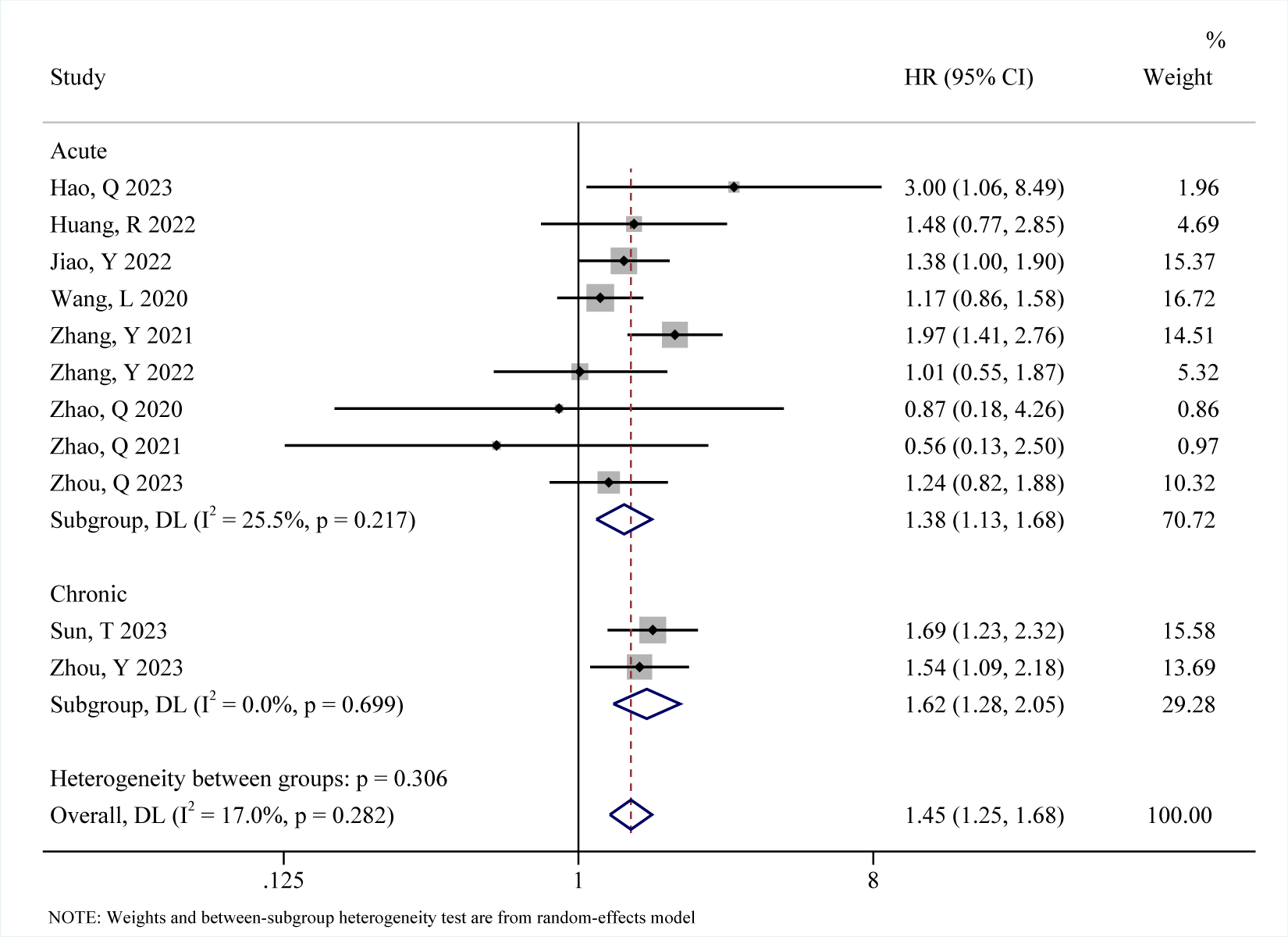


**Suppl 4.** All-cause subgroup.

Supplement: Suppl 4 — All-cause mortality. [file jocmr-18-06-407-s004.docx]

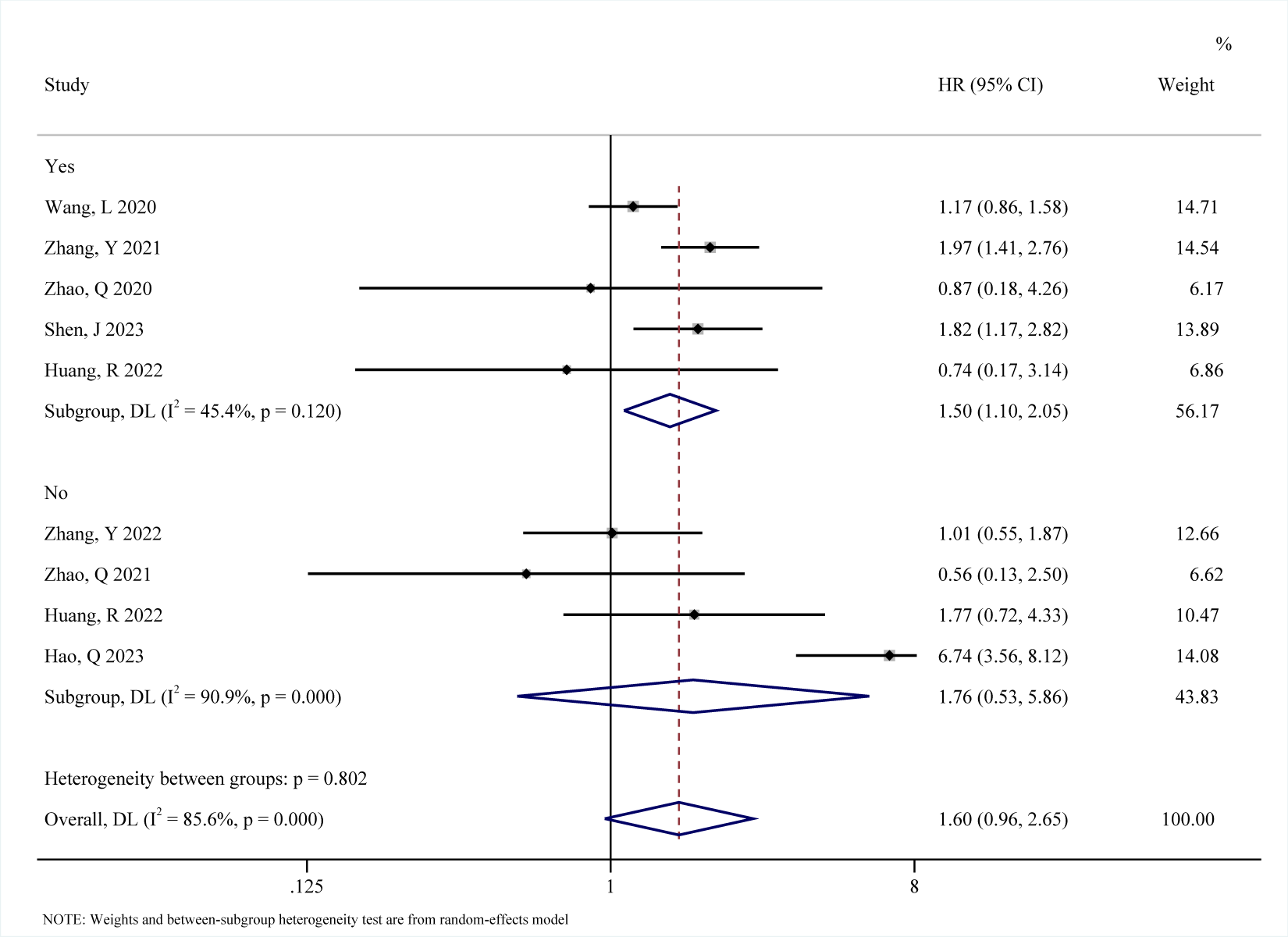


**Suppl 5.** All-cause diabetes subgroup.

Supplement: Suppl 5 — CVD mortality. [file jocmr-18-06-407-s005.docx]
